# Effect of solid substrates on the production and germination of *Beauveria bassiana* (Balsamo) Vuillemin, and their infectivity against the malaria vector, *Anopheles stephensi* (Diptera: Culicidae)

**DOI:** 10.1186/s12936-026-05863-7

**Published:** 2026-04-11

**Authors:** Renuka Siddaramegowda, Vani H. Chalageri, Alex Eapen

**Affiliations:** 1 ICMR-National Institute of Malaria Research Field Unit, Bengaluru, Karnataka India; 2 ICMR-National Institute of Malaria Research Field Unit, Chennai, Tamil Nadu India; 3 Academy of Scientific and Innovative Research (AcSIR), Ghaziabad, India; 4 Central Research Laboratory, MAHER-Meenakshi Medical College Hospital and Research Institute, Kanchipuram, Tamil Nadu India

**Keywords:** *Anopheles stephensi*, Malaria, *Beauveria bassiana*, Vector control, Entomopathogenic fungi

## Abstract

**Background:**

The growing insecticidal resistance in vector mosquitoes is alarming and calls for an alternative, safe, and eco-friendly control strategy. As an alternative, entomopathogenic fungi (EPF) have gained attention. Hence, the present study evaluated different solid substrates to understand the nutrient requirements of EPF for conidial production and their infectivity against the vector mosquito.

**Methods:**

Eleven solid substrates were tested for the conidial production of PDBC-Bb5a. The conidial load per gram of substrate was calculated using the spread plate technique. The conidial germination rate at 8, 16, and 24 h after incubation was recorded. The conidial infectivity against *Anopheles stephensi* was assessed with the WHO cone bioassay tests, exposing mosquitoes to PDBC-Bb5a (1 × 10^7^ conidia/mL) treated cement and mud panels for 30 min. Four replicates, each containing 20 mosquitoes, were maintained for each substrate and the control group. Mosquito mortality was recorded over 10 days. ANOVA was performed to examine the difference between conidia produced on different solid substrates, and the Wilcoxon signed-rank test was used to compare germination rates at 8 versus 16 h of incubation. The Kaplan–Meier test was undertaken to represent the survival function.

**Results:**

The highest number of PDBC-Bb5a conidia was produced on white rice (6.8 × 10^5^ CFU g^−1^), followed by sorghum and broken wheat (*p* < 0.05). Further, the highest germination rate was achieved from conidia produced on white rice (37%) and broken wheat (56%) at 8 and 16 h of incubation, respectively. An increase in germination rate overtime was observed on white rice (37.0 to 54.3% at 8 and 16 h) and broken wheat (36.3% at 8 h and 56.0% at 16 h), yet there was no significant difference among the substrates. The conidia produced on white rice recorded 90% and 86.25% of mortality with 5 (χ2 = 200.32; df = 5; *p* < 0.01) and 7 (χ2 = 200.32; df = 5; *p* < 0.01) median survival times of *An. stephensi* on cement and mud panels, respectively. However, barley observed 51.25 and 47.50% mortality with 10 days of median survival time on cement and mud panels, respectively.

**Conclusion:**

The study proved that white rice was a better substrate for the production of *B. bassiana.* Further multicentric studies are needed to test in different eco-geographical areas to assess the efficacy and persistence in varied environmental conditions before considering it in vector control programs.

## Background

Malaria is a vector-borne disease, transmitted by the *Anopheles* mosquito [1]. Vector control mainly relies on chemical insecticides belonging to the classes carbamate, organochlorine, organophosphate, and pyrethroid. Due to the overuse of currently available insecticides, vector mosquitoes have gained resistance [2–4]. As an alternative and an eco-friendly method, entomopathogenic fungi (EPF) such as *Metarhizium* and *Beauveria* have been reported to be potential against vector mosquitoes [5–9].

Most of the studies have used EPF dry conidia or blastospores produced on synthetic cultural medium to check the efficacy against vector mosquitoes [8, 10–15]. Different techniques, such as fungal-based silver nanoparticles (AgNPs) [16], oil formulations [17, 18], attractive baits [19], etc., were employed to enhance the fungi’s efficacy against vector mosquitoes. However, there is a lack of studies exploring solid substrates to enhance the EPF infectivity against the malaria vector, *Anopheles stephensi*. The selection of the substrate for the conidial production plays a significant role. The nutrient content of the substrate, particularly the carbon and nitrogen (C/N) ratio, modifies the conidial yield, viability, desiccation tolerance, and the infectivity of the fungi towards specific targets [20–22]. The solid substrate fermentation technique is extensively used for natural processes for EPF production. Starch-rich agricultural products have been widely used for mass production due to their easy degradation through hydrolytic enzymes. [23, 24]. The use of cereals for conidial production is a conventional method in Latin America. During the 1970 s, *Metarhizium anisopliae* was mass-produced on grains and applied to an area of 100,000 ha/year to control the spittlebug *Mahanarva posticata* Stal (Auchenorrhyncha: Cercopidae) in a sugarcane field in Brazil [25]. The suitable substrate should not only increase the conidial production but also enhance the virulence of the conidia.

*Beauveria bassiana* has been reported to be potentially effective against *An. stephensi* in our previous study [7]. However, a scarcity of appropriate and economically feasible substrates for the selected isolate, and so the study has been planned to screen for suitable solid substrates for conidial production. Since the C/N ratio of the substrates is one of the crucial parameters to increase the conidial production [26], the substrates were selected based on their nutritional sources. Broken wheat, brown rice, and barley have been selected based on their high C/N ratio around 20–30:1 [9]. The lesser C/N content substrates, such as sorghum, horse gram, green gram, black gram, groundnut, sesame, and finger millet, were selected because EPF has the potential to produce more virulent conidia under nutrient stress conditions [27]. White rice has been selected as one of the solid substrates since it is a highly preferred substrate for *B. bassiana* [28].

## Methods

### Entomopathogenic fungi

In the present study, the *B. bassiana* isolate, PDBC-Bb5a, was selected based on its pathogenicity against *An. stephensi* reported in our previous study [7]. PDBC-Bb5a was procured from ICAR-National Bureau of Agricultural Insect Resources (ICAR-NBAIR), Bengaluru. PDBC-Bb5a was originally isolated from a coffee berry borer (*H. hampei*) cadaver, maintained on Sabouraud Dextrose Yeast extract Agar (SDYA) cultural medium and stored at − 20 °C for further use.

### Solid substrates

A total of eleven solid substrates have been selected for the current study based on their C/N contents and the available surface area for fungal growth. The substrates included white rice, broken wheat, sorghum, brown rice, horse gram, green gram, black gram, barley, groundnut, sesame, and finger millet for the production of PDBC-Bb5a was selected.

### Preparation of fungal inocula

PDBC-Bb5a isolate was grown in a 100 mL conical flask containing 25 mL of Sabouraud Dextrose Yeast extract Broth (SDYB) medium. The conical flask was inoculated with a loop full of PDBC-Bb5a culture. The flask was then incubated at 25 ± 1 °C in a rotary shaker incubator (Orbitek 0I02DCB4, Sl.No: 2013400095, Scigenics Biotech Pvt Ltd., India) at 150 rpm for five days. After incubation, the conidial concentration in the shaker culture was adjusted to the desired conidial concentration of 10^8^ conidia/mL using a Neubauer-improved haemocytometer. The prepared conidial suspension was used as an inoculum for substrate inoculation [29].

### Solid substrates preparation and inoculation

Approximately 100 g of each solid substrate (three replicates/substrate) was washed thoroughly with water and soaked for 1–8 h with distilled water in polypropylene bags. After soaking, the excess water was drained off. The bags were plugged with non-absorbent cotton and sterilized in an autoclave at 121 °C for 20 min. After sterilization, the substrates were cooled and then inoculated with 1 mL (10^8^ conidia/mL) of the above-prepared PDBC-Bb5a conidial suspension. The bags were sealed and incubated at 25 ± 1 °C for 15 days [29]. Each bag was checked every day for any contamination, and the inoculated substrate was mixed for proper distribution of the fungus on the solid substrate and to avoid clump formation.

### Estimation of conidial load per gram of substrate

Following 15 days of incubation, the conidial load per gram of conidiated substrate was estimated using a serial dilution plating technique. One gram (from each replicate) of 15-day-old conidiated substrate was weighed under aseptic conditions and transferred into a sterilized 25 mL screw cap tube containing 9 mL of sterile distilled water. The solution was shaken vigorously using a vortex shaker to suspend all the conidia in the water. Once all the conidia were suspended, a dilution series of 10^1^ to 10^7^ was carried out. Around 0.1 mL of sample was taken from 10^3^ to 10^5^ serially diluted tubes and was plated into their respective SDYA plates using the pore plate method. Three replicates (plates) were maintained for each dilution. The plates were incubated at 25 °C for five days, after which the colony-forming units (CFUs) were counted. The conidial load per gram of substrate was calculated [30].

### Germination rate of conidia produced on different solid substrates

The germination tests for the PDBC-Bb5a conidia produced on different solid substrates were undertaken at three different incubation-time intervals: 8, 16, and 24 h of incubation. Three sets of SDYA plates were prepared and were inoculated with 0.1 mL of the respective conidial suspension using the spread plate technique. The first set of plates was incubated for 8 h, the second set for 16 h, and the third set was incubated for 24 h at 25 °C ± 1 °C in an incubator. Three replicates (plates) were maintained for each incubation period. After incubation, the agar plate lid was removed, a coverslip was placed on the surface of the agar, and conidia were examined under fluorescence microscopy (Carl Zeiss Microimaging GmbH-37081, Germany, Serien-Nr.: 3,517,002,452, Axio Imager. A1) with a magnification of 40x. To determine the percentage of conidial germination, the conidia with the germination tube that were at least double the length of individual conidia were counted. A minimum of 200 conidia were examined, and the percentage of germination was calculated [31].

### Culture and maintenance of *Anopheles stephensi* mosquitoes

Field collected *An. stephensi* mosquitoes were identified using the standard taxonomic keys at ICMR-National Institute of Malaria Research, Field Unit (NIMRFU), Bengaluru, India [32]. The mosquitoes were maintained under standard insectary conditions of 25 ± 2 °C temperature, 80 ± 10% relative humidity (RH) according to the WHO standard protocol [33]. 14 h:10 h light:dark photoperiod was maintained. The larvae were maintained in plastic rearing trays and were fed on Tetramin^®^ baby fish food until pupation. Pupae were collected every day and immediately transferred into an adult mosquito cage of size 2 feet × 2 feet. The emerged adult mosquitoes were maintained on a 10% glucose solution. Live Swiss albino mice, after obtaining Institutional Ethical Clearance (IEC), were used as a blood source to feed the female mosquitoes to facilitate egg development.

### Infectivity of PDBC-Bb5a conidia produced on different solid substrates against *Anopheles stephensi*

Among the eleven solid substrates tested for the production of the conidia, only five substrates, such as white rice, broken wheat, brown rice, barley, and sorghum, supported the growth of PDBC-Bb5a. Hence, the conidia produced on these substrates were used to study the infectivity of the conidia against *An. stephensi*, through the WHO cone bioassay tests.

### Preparation of inocula

The *B. bassiana* isolate PDBC-Bb5a was cultured on white rice, broken wheat, brown rice, barley, and sorghum as described above. One gram of conidiated substrate was transferred into the respective beaker containing 10 mL of sterile distilled water. The beakers were shaken vigorously using a vortex shaker (REMI, Maharashtra, India) to release the conidia into the solution. Later, the conidial suspension was filtered through a sterilized No.1 Whatman filter to obtain a hyphal-free conidial suspension. The desired conidial concentration of 1 × 10^7^ conidia/mL was adjusted using a Neubauer-improved haemocytometer.

### Preparation of panels

Cement and mud panels were prepared following our previous study [7]. In brief, cement panels were prepared using cement and sand with a ratio of 1:5. The mud panels were prepared using locally collected red soil at ICMR-NIMRFU campus, Bengaluru, India. The panels were measuring around 30 × 30 cm^2^ with a thickness of 1 cm. Panels were dried at room temperature for 4 weeks. Approximately 20 mL of the respective conidial suspension was sprayed on each panel using a conventional hand-held spray gun having a nozzle size of 1.4 to 2.5 mm. Treated panels were kept dry for 24 h, after which they were used for the WHO cone bioassay tests.

### WHO cone bioassay tests

**Fig. 1:**
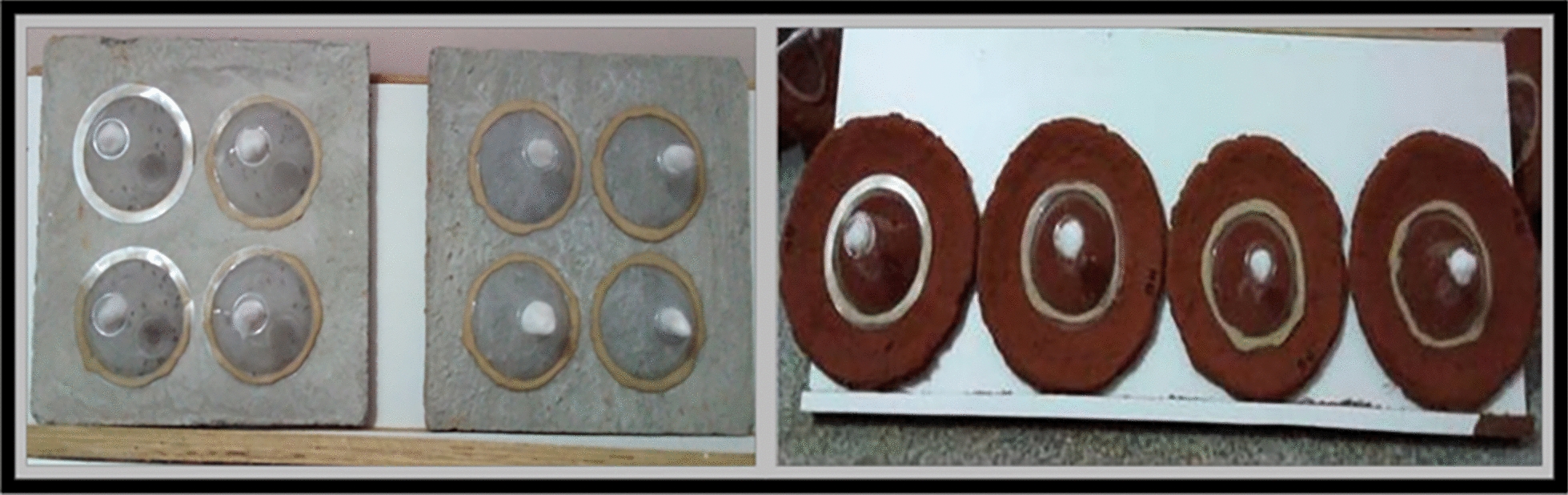
WHO cone bioassay tests on PDBC-Bb5a-treated (conidia produced on different solid substrates) cement and mud panels with the conidial concentration 1 × 10^7^ conidia/mL against *Anopheles stephensi* mosquitoes

The cone bioassays were conducted using WHO-standard cones and exposure procedures (Fig. 1) [34]. Three to five-day-old, sugar-fed, adult female mosquitoes were used for the study. Four replicates were maintained for all five treatments (conidia produced on five solid substrates) and the control. 20 mosquitoes per replicate were maintained, including the control. The adult female mosquitoes were exposed to the fungal-treated respective panels for 30 min. Then, treated mosquitoes were transferred to holding cups and provided with a 10% glucose solution. The observation was recorded at 24 h intervals for 10 days. Dead mosquitoes were removed during the observation, the surface was sterilized, and they were placed on moist, sterilized Whatman filter paper to facilitate the mycosis. The mosquito mortality rate was calculated 10 days after treatment.

### Data and statistical analysis

The control mortality was adjusted using Abbott’s formula [35] wherever applicable. Before the ANOVA, assumptions of normality were evaluated through the Shapiro–Wilk test. White rice (*p* = 0.927), brown rice (*p* = 0.927), barley (*p* = 0.537), and sorghum (*p* = 0.702) met the normality assumption (*p* > 0.05), but wheat substrate showed a deviation from normality (*p* < 0.05) with *p* = 0.00. Since the sample size per treatment (*n* = 3) was less and the Levene’s test indicated no significant differences in error variances among substrates (*F *(4,10) = 1.108, *p* = 0.405), conidial production data were analyzed using one-way ANOVA, followed by Tukey’s HSD for pairwise comparison (*p* < 0.05). The Wilcoxon signed-rank test was performed to compare the germination rate at 8 versus 16 h of incubation. The one-way ANOVA, followed by Tukey’s HSD for pairwise comparison (*p* < 0.05), was performed for mean comparison of germination rate. The arcsine transformation was applied to all percent mortality data. The mean percent mortality data were compared using the Tukey HSD test (*p* ≤ 0.05). The adult survival curve was obtained using the Kaplan–Meier estimator. Significant differences between the infectivity of the conidia produced on different solid substrates were estimated using the Log Rank (Mantel–Cox) test. All statistical analyses were conducted using SPSS version 16.0 statistics program [36].

## Results

### Estimation of conidial load per gram of substrate

**Fig. 2:**
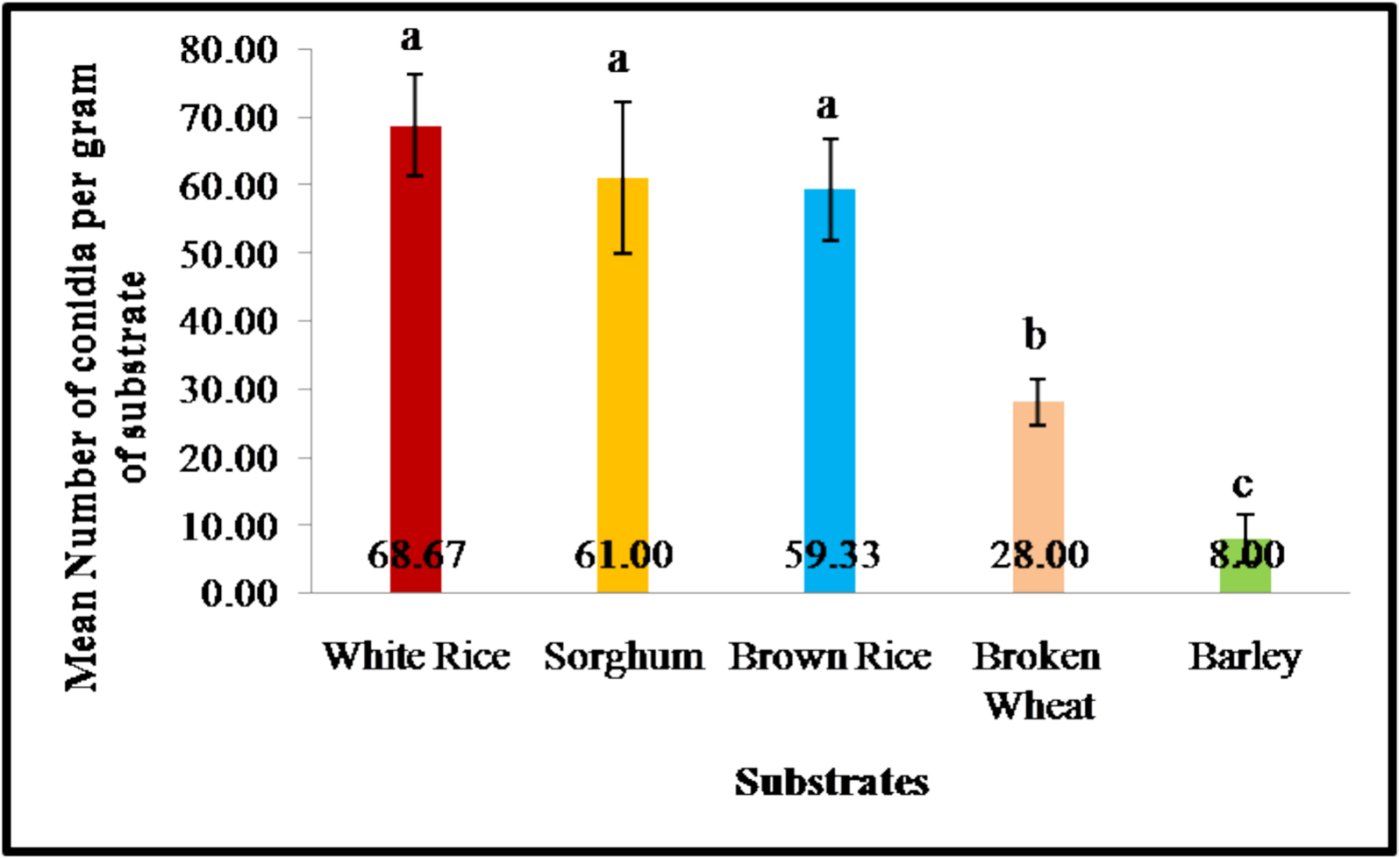
Mean number of PDBC-Bb5a conidia produced on one gram (10^5^ dilution) of solid substrate after 15 days of incubation. The error bars represent the standard error (n = 3), and the means with the same alphabet are not significantly different (Tukey HSD, p > 0.05)

The conidial production of the PDBC-Bb5a isolate varied significantly across the solid substrates tested. Among the eleven substrates evaluated, only five substrates, such as white rice, broken wheat, brown rice, barley, and sorghum, have supported fungal growth (Fig. 2). Out of which, white rice produced the highest mean conidial count at 68.67 × 10^5^ CFU · g^–1^of substrate, followed by the sorghum (61 × 10^5^ CFU · g^–1^) and the brown rice (59.33 × 10^5^ CFU · g^–1^). Since there was an overlap in the 95% CI of white rice (59.36–77.97), Sorghum (51.69–70.30) and brown rice (50.02–68.63), there were no significant differences among them. Broken wheat produced a moderate level of conidia (M = 28.00; 95% CI = 18.69–37.31). The negative value of barley at the lower bound of CI (− 1.31 to 17.31) indicated that there is low reliability in conidial yield on this substrate. These results indicated that white rice, followed by sorghum and brown rice, are suitable substrates for the production of PDBC-Bb5a conidia.

### Germination rate of conidia produced on different solid substrates

The germination of conidia produced on five solid substrates was assessed at 8, 16, and 24 h of incubation. The germination rate at 24 h after incubation was not recorded due to excessive fungal hyphal growth, and the overlapped hyphae interfered with the observation. Hence, 24 h of observation data were omitted from the results. However, the 8 and 16 h data were used for statistical analysis and interpretation.

**Fig. 3:**
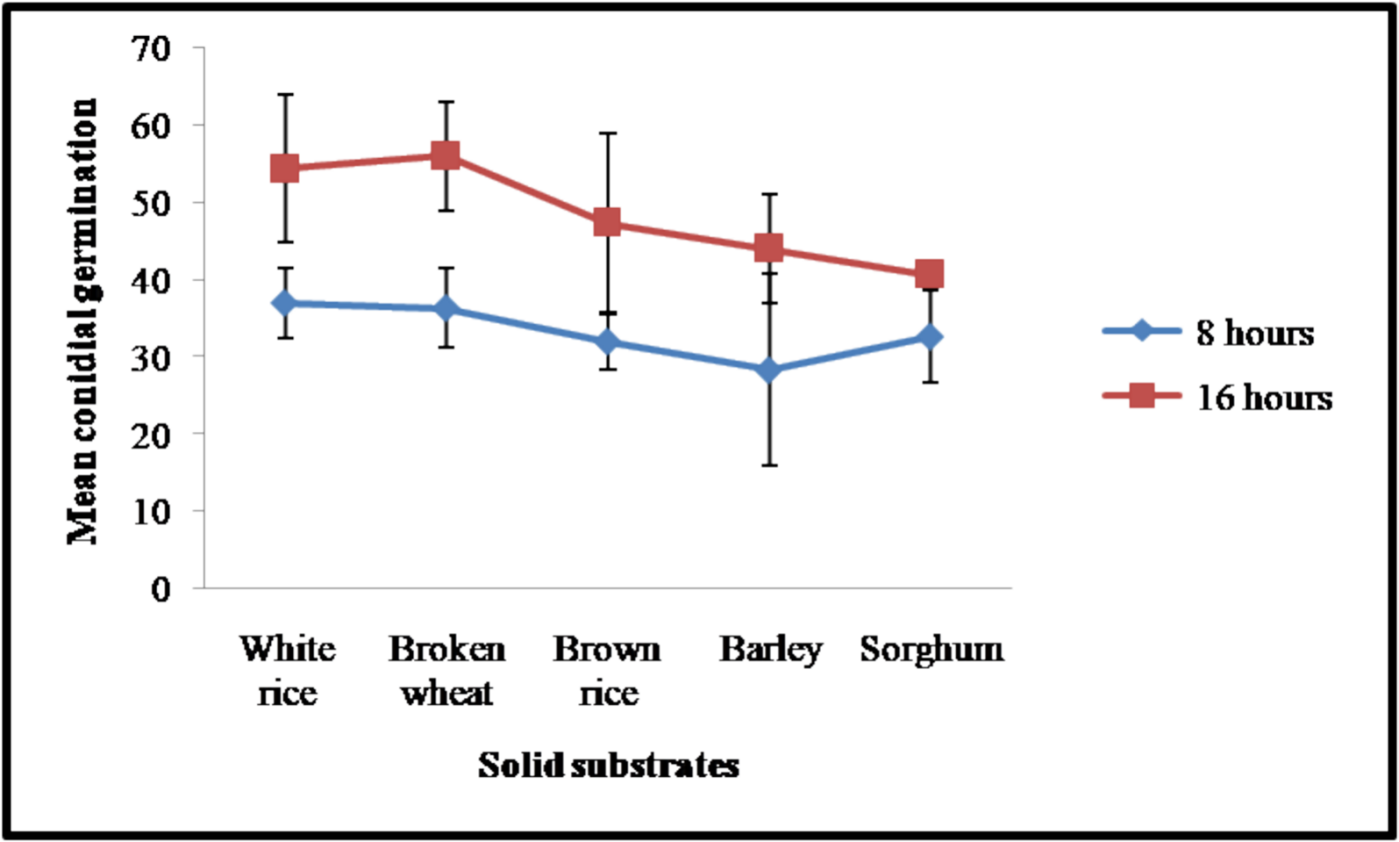
The mean germination rate (%) ± standard error (n = 3) of PDBC-Bb5a conidia produced on different solid substrates at 8 and 16 h of incubation. The data are represented as the mean germination percent. The error bars represent the standard error at a significance level of 0.05

**Fig. 4:**
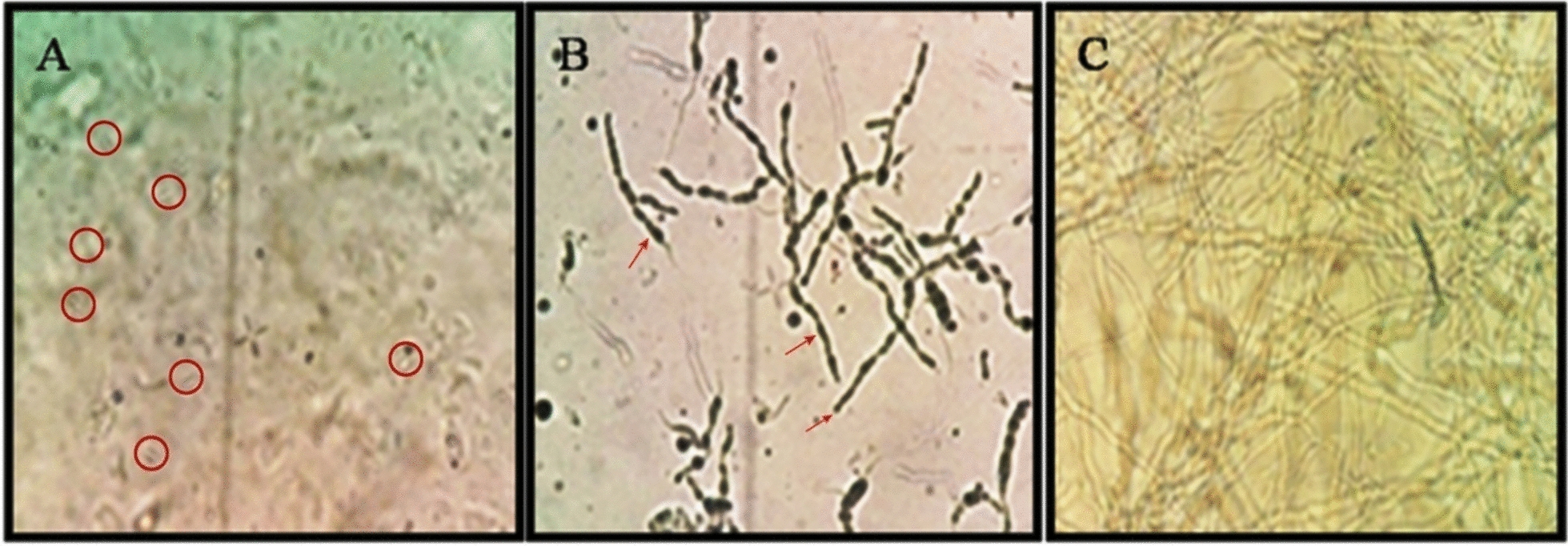
Microscopic view at 40X. PDBC-Bb5a conidial germination at different time intervals. **A** Red colored circles showing germinated conidia at 8 h of incubation. **B** Red colored arrows indicate the fungal growth at 16 h of incubation, and **C** shows completely grown fungal hyphae at 24 h of incubation

The Levene’s tests indicated that the assumption of homogeneity of variance was met at *F *(4,10) = 2.029, *p* = 0.166 (8 h) and *F*(4,10) = 1.157, *p* = 0.385 (16 h).The conidia produced on white rice and wheat recorded higher germination rates compared to barley and sorghum. However, the substrates did not significantly affect the germination rate among the substrates at both 8 h (*F *(4,10) = 0.748, *p* = 0.581, partial η^2^ = 0.230) and 16 h (*F*(4,10) = 1.999, *p* = 0.171, partial η^2^ = 0.444) incubation intervals. The Tukey HSD homogeneous subsets confirmed that none of the differences were statistically significant at 8 h (*p* = 0.582) and 16 h (*p* = 0.213) incubation intervals. But, the conidial germination rate significantly increased overtime on a few substrates (Figs. 3, 4). Increased germination rate was observed from 37.0 to 54.3% at 8 and 16 h, respectively, for white rice. Similarly, broken wheat also recorded an increased germination rate overtime (36.3% at 8 h and 56.0% at 16 h). Brown rice (32.0% at 8 h and 47.3% at 16 h) and barley (28.3% at 8 h and 44.0% at 16 h) were not statistically significant. Sorghum recorded a poor increase in germination from 32.7 to 40.7% at 8 and 16 h of incubation, respectively. Overall, the Wilcoxon signed-rank test yielded a Z-value of − 2.023 with a *p*-value of 0.043, indicating a significant difference between the intervals. The Z-value showed a negative value (− 2.023^a^) with a *p*-value of 0.043, confirming that the germination rate at 16 h after incubation is significantly different from that at 8 h of incubation.

### Infectivity of PDBC-Bb5a conidia produced on different solid substrates against *Anopheles stephensi*

**Fig. 5:**
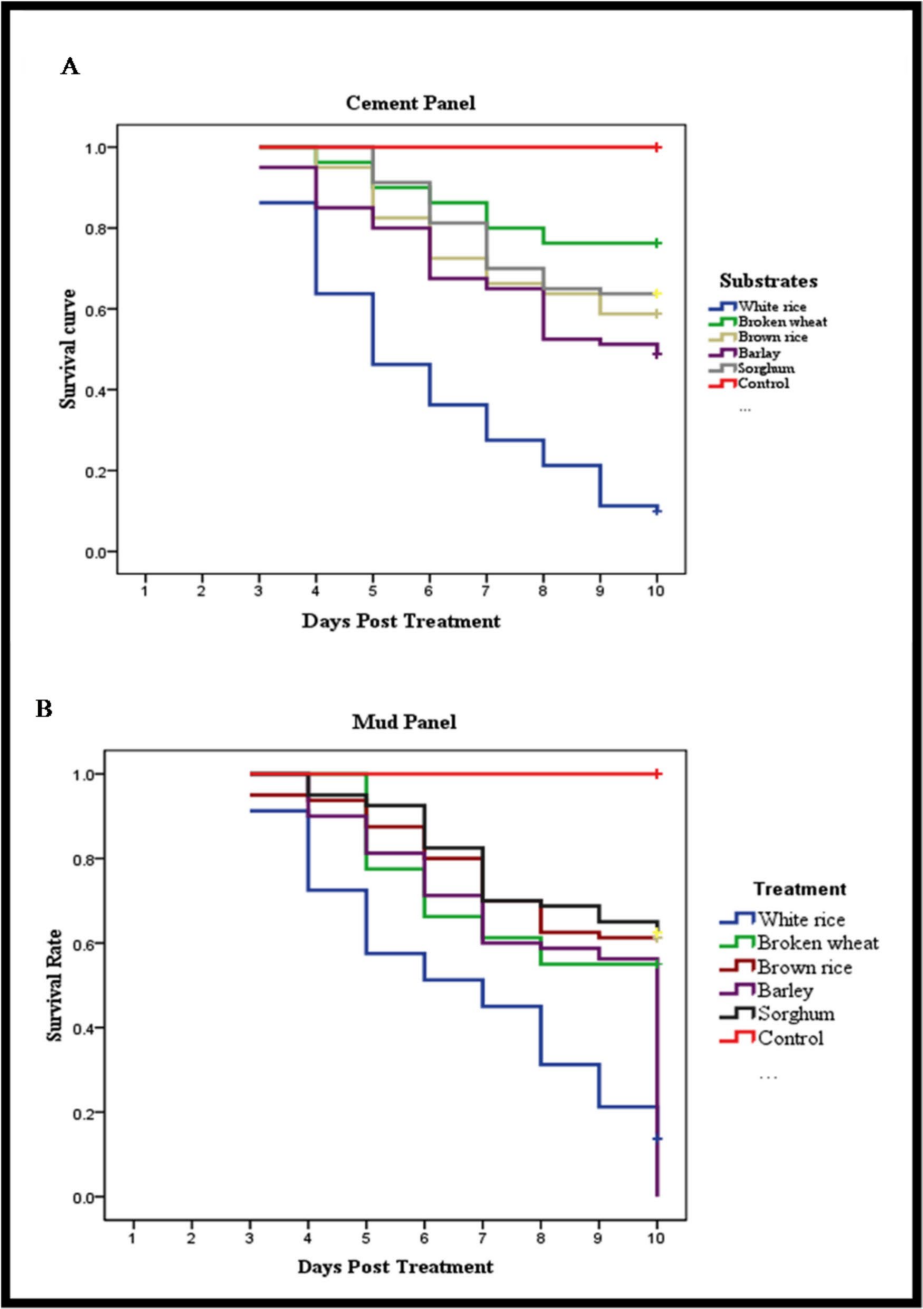
Survival rate of adult female mosquitoes, *Anopheles stephensi* exposed to PDBC-Bb5a with the conidial concentration 1 × 10^7^ conidia/mL produced on different solid substrates, white rice, broken wheat, brown rice, barley, and sorghum. **A** Data are represented for the cement panel, and **B** Data are represented for the mud panel

The substrates that supported PDBC-Bb5a growth significantly affected the infectivity of the conidia (Table 1). The PDBC-Bb5a conidia produced on white rice were found to be more virulent than the conidia produced on other substrates. Conidia produced on white rice resulted in 90% mortality (CI = 78.75—101.25) of *An. stephensi* mosquitoes on the cement panels and 86.25% mortality (CI = 76.24—96.26) on the mud panels. Regarding the median survival time (MST) of *An. stephensi*, the lowest MST of 5 days on cement panels (χ2 = 200.32; df = 5; *p* ≤ 0.01) and 7 days on mud panels (χ2 = 200.32; df = 5; *p* ≤ 0.01) were recorded for conidia produced on white rice. This was followed by conidia produced on barley, which caused 51.21% of the mortality of *An. stephensi* on cement panels with 10 days of MST (Fig. 5). However, conidia produced on sorghum were found to be less virulent against *An. stephensi* mosquitoes on both cement and mud panels (23.75 and 37.50%), respectively.

**Table 1:** Percent mortality of adult female mosquitoes, *Anopheles stephensi,* exposed to PDBC-Bb5a with the conidial concentration 1 × 10^7^ conidia/mL treated cement and mud panels through the WHO cone bioassay tests. PDBC-Bb5a conidia were produced on white rice, broken wheat, brown rice, barley, and sorghum

Mean percent mortality within 10 days						
Solid substrates	Cement panels			Mud panels		
	Mean mortality	SD	95% CI	Mean mortality	SD	95% CI
White rice	90.00^a^	7.07	78.75–101.25	86.25^a^	6.29	76.24–96.26
Broken wheat	23.75^d^	4.78	16.13–31.37	45.00^bc^	7.07	33.75–56.25
Brown rice	42.50^bc^	10.40	25.94–59.06	38.75^c^	8.53	25.16–52.34
Barley	51.25^b^	13.76	29.34–73.16	47.50^b^	2.88	42.91–52.09
Sorghum	36.25^ cd^	10.30	19.85–52.65	37.50^c^	5.00	29.54–45.46
Control	0.00^e^	0.00	0.00	0.00^d^	0.00	0.00

^*^The means with the same alphabet are not significantly different

## Discussion

The current study emphasizes the importance of selecting a suitable substrate for the production of virulent conidia for the management of vector mosquitoes. The selection of substrates depends not only on fungal species, but also on isolates [37]. The white rice is considered a suitable substrate for the production of *Beauveria* [28, 30, 38] due to its suitable carbon and nitrogen proportion, moisture holding capacity, and surface area [39, 40]. Similarly, the present study also observed that white rice, followed by sorghum and brown rice, yielded more conidia. But, in contrast, few studies have reported that wheat supports better conidial yield [37, 41]. Nevertheless, broken wheat and barley recorded lower conidial production. It might be due to their nutrient imbalance. For instance, broken wheat may have a high amount of starch and too little nitrogen, which may affect the conidial production [42]. Further, barley is a dense substrate likely to hold high moisture content compared to white rice, which creates anaerobic conditions and could affect the conidial production [43]. The surface area of the substrate also plays a major role in conidial production. Broken wheat is prone to forming clumps after sterilization due to high moisture content. This promotes uneven growth of the fungi and lowers the conidial production [44]. However, some of the tested substrates, such as horse gram, green gram, black gram, groundnut, sesame, and finger millet, did not support fungal growth. Black gram, green gram, groundnut, and ragi were suitable for the growth of *M. anisopliae* [45]. EPF require specific nutrients such as carbon, nitrogen, minerals, and vitamins in balanced quantities for their growth. *Beauveria* utilizes carbon and nitrogen sources for germination and hyphal development, respectively, while some of the *M. anisopliae* strains do not utilize glucose as a nutrient for their conidial germination [46]. Thus, the findings indicate the significant differences among the substrates in their potential to produce conidia and their infectivity.

Furthermore, studies have revealed that the nutritional content, moisture content, and physical properties of substrates directly influence conidial germination and infectivity. Conidia produced on different substrates showed varied germination rates at different time intervals. The highest germination was observed in conidia produced on rice, followed by broken wheat at 8 h after incubation. But the germination rate was higher in conidia produced on broken wheat than in rice at 16 h after incubation (Fig. 3). Interestingly, no significant changes in the germination of conidia produced on different substrates were reported [47]. The nutrient composition of growth substrates influences fungal infectivity [48–50] by altering the spore quality, such as germination, metabolic activities, and enzyme activities of the conidia [51–54]. Slow-germinating fungal propagules are not effective biocontrol agents. Although fungal blastospores have been considered as a potential alternative compared to conidia due to their vigorous germination [55]. Conidia produced on broken wheat were shown to be less virulent (51.25% (cement) and 47.50% (mud)) against *An. stephensi*, even when the germination rate was high at 16 h after incubation. It is interesting to note that 90% mortality was observed in *An. stephensi* for conidia produced on white rice. This result indicates that the conidia produced on white rice are more effective against *An. stephensi* than those produced on synthetic SDYA medium [7]. Similarly, *B. bassiana* grown on Potato Dextrose Agar (PDA) and Czapek-Dox medium (CZA) mycological media recorded higher efficacy than conidia grown on C/N-rich medium, SDYA [56]. Maldonado et al. observed higher submerged spore yields of *B. bassiana* in casamino, but it was virulent to *Ae. aegypti* only when grown on soybean flour [57].

The present study revealed the potential of PDBC-Bb5a growth on different solid substrates and their infectivity against *An. stephensi* under controlled conditions. The fungus may reduce its infectivity due to abiotic and biotic stress, and also, the fungal infectivity and germination rate under field conditions may vary. Hence, further multicentric field trials are required to test the potential of PDBC-Bb5a to consider it in the vector control programs.

## Conclusion

The current study revealed that the efficacy of PDBC-Bb5a is closely related to the type of substrate. Conidia produced on white rice recorded the highest production, germination, and mortality of *An. stephensi* with a shorter median survival time on both mud and cement panels. This highlights not only the significance of the conidial quantity but also quality attributes like viability, persistence, and infectivity. The lower efficacy of conidia produced on sorghum, even with their high conidial production, indicates dissociation between yield and efficacy. This underlines the need for further bioassay-based studies for quality control in the development of biopesticides against vector mosquitoes. The substrate selection should be prioritized for both conidial production and its quality, such as germination rate and infectivity. However, white rice remained the best choice of substrate for the production of PDBC-Bb5a based on evaluated parameters such as conidial yield, germination, and infectivity. Since it was an initial screening, more studies could evaluate substrate combinations with different moisture content to improve the conidial efficacy against *An. stephensi*. Overall, the current findings provide critical insights into improving the production and efficacy of PDBC-Bb5a conidia. Hence, further multicentric studies are needed to test in different eco-geographical conditions to assess the efficacy and persistence in varied environmental conditions before considering it in vector control programs as an alternative to public health chemical pesticides.

## Limitations of the study

The current study did not maintain the same moisture content of the substrates. Since substrates have varied water-holding capacity, it would affect the nutrient availability for the EPF if the substrate’s moisture level were standardized. The study had to omit the data on germination rate at 24 h of incubation due to the overgrowth of the fungal conidia. The control set for conidial yield and germination was considered because the study aimed to select suitable solid substrates for the mass production of EPF. Hence, a comparison was made among the substrates. As the current study is a laboratory-based evaluation, the persistence and efficacy of the fungi were not assessed in field conditions.

## Data Availability

The data presented in this study are available on request from the corresponding authors.
